# Large area micropatterning of cells on polydimethylsiloxane surfaces

**DOI:** 10.1186/1754-1611-8-24

**Published:** 2014-10-24

**Authors:** Mahmoud E Moustafa, Venkat S Gadepalli, Ahmed A Elmak, Woomin Lee, Raj R Rao, Vamsi K Yadavalli

**Affiliations:** Department of Chemical and Life Science Engineering, Virginia Commonwealth University, Richmond, VA 23284 USA

**Keywords:** Cell micropatterning, Poly (dimethyl siloxane), Photolithography, Poly (ethylene glycol)

## Abstract

**Background:**

Precise spatial control and patterning of cells is an important area of research with numerous applications in tissue engineering, as well as advancing an understanding of fundamental cellular processes. Poly (dimethyl siloxane) (PDMS) has long been used as a flexible, biocompatible substrate for cell culture with tunable mechanical characteristics. However, fabrication of suitable physico-chemical barriers for cells on PDMS substrates over large areas is still a challenge.

**Results:**

Here, we present an improved technique which integrates photolithography and cell culture on PDMS substrates wherein the barriers to cell adhesion are formed using the photo-activated graft polymerization of polyethylene glycol diacrylate (PEG-DA). PDMS substrates with varying stiffness were prepared by varying the base to crosslinker ratio from 5:1 to 20:1. All substrates show controlled cell attachment confined to fibronectin coated PDMS microchannels with a resistance to non-specific adhesion provided by the covalently immobilized, hydrophilic PEG-DA.

**Conclusions:**

Using photolithography, it is possible to form patterns of high resolution stable at 37°C over 2 weeks, and microstructural complexity over large areas of a few cm^2^. As a robust and scalable patterning method, this technique showing homogenous and stable cell adhesion and growth over macroscales can bring microfabrication a step closer to mass production for biomedical applications.

**Electronic supplementary material:**

The online version of this article (doi:10.1186/1754-1611-8-24) contains supplementary material, which is available to authorized users.

## Background

The development of methods to spatially direct cell growth in two and three dimensions is a fundamental challenge for *in vitro* research and simulating *in vivo* cellular microenvironments [1, 2]. Beyond applications in tissue engineering and microarray technologies, precisely controlling the location of cells has potential in furthering our understanding of fundamental cellular processes [3–5]. Precise regulation of cell response and fate can reveal insights into intercellular interactions and cues [6]. By integrating microfabrication strategies, it is possible to form efficiently controlled cell cultures, or lead to hierarchical organization as tissues and organs [7]. Typically, spatial control has been achieved by creating well-defined physical or biochemical barriers, or cell-adhesive regions to encourage specific attachment. Conversely, controlling non-specific adsorption in regions where cell growth is not desired provides a similar effect. Nevertheless, maintaining microscale precision and uniformity over large areas (cm), a characteristic of live tissues, remains a challenge for translation to application.

Various synthetic and natural materials are used as substrates for cell growth and differentiation [8]. As an alternative to rigid polystyrene and glass surfaces, poly (dimethylsiloxane) (PDMS) is a versatile polymer that has been widely used as an elastic, stretchable, cellular substrate in the form of microfluidic channels, microwells, and micro- and nano-pillars [3, 9, 10]. PDMS possesses unique advantages including optical transparency, biocompatibility, flexibility, tunable mechanical properties, oxygen permeability, durability and low cost [11]. However, the surface of PDMS is highly hydrophobic (contact angle ~105°) which tends to result in the non-specific adsorption of proteins and other biomolecules required for cell attachment and growth [12, 13]. Surface modification is therefore required for effective spatial regulation of cells. Typically, cells have been grown on PDMS substrates without specific spatial control [11, 14]. On the other hand, micropatterning strategies on PDMS have usually involved microcontact printing (μCP) to form high resolution cell-adhesive patterns [15, 16]. Surface modification using plasma oxidation of PDMS to increase hydrophilicity followed by surface functionalization has also been reported [17]. However, these physisorption approaches are typically non-covalent in nature, confined to small areas (mm), or do not present adequate physical barriers for cellular growth, making them short-lived [18]. Forming physico-chemical barriers can confine cells to adhesive regions, while allowing growth over extended periods of time and over large areas. Adapting photolithography to form geometrically distinct barriers for specific cell attachment provides the ability to easily fabricate high resolution patterns over large areas [5, 19].

Surface modification using hydrophilic and neutrally charged polymers, in particular homo and hetero-functional polyethylene glycol (PEG) hydrogels, has been extensively used to repel non-specific protein adsorption and guide cell attachment [20–24]. Using PEG on PDMS therefore provides a means to precisely direct cell adhesion. However, covalently attaching PEG to PDMS has been difficult. Photo-induced grafting for surface modification of PDMS was first demonstrated using acrylic acid (PAA), acrylamide and polyethylene glycol methacrylate (PEG-MA) monomers [25]. However, both PEG-MA and PAA are not optimal owing to surface charges and gradual loss of hydrophilicity resulting in eventual cell adhesion. Further, PEG-MA yields fragile patterning and cannot withstand physiological or microfluidic shear stresses [26]. Photo-induced graft polymerization using polyethylene glycol diacrylate (PEG-DA) was used to micropattern PDMS, which is effective with long-lasting hydrophilic properties and stable patterns over 2 months [10]. The micropatterned-PEGDA-grafted PDMS was applied to protein adsorption and cell adhesion. However, a reliable strategy to form stable, micro and macroscale patterns over large areas on different PDMS compositions is still challenging.

One of the advantages of using PDMS is its tunable mechanical nature. By controlling the ratio of monomer to crosslinker, the stiffness of the underlying substrate can be altered. This in turn has a great influence on the cell growth on the surface [27]. For instance, our group and others have previously shown that human embryonic stem cell proliferation can be affected by varying the stiffness [28, 29]. Characteristically, exposure of cell-binding motifs differ based on the nanoscale surface stiffness, reflecting changes in cell behavior [30]. In this work, we investigate the photopatterning of PEG-DA hydrogels that can be used on PDMS surfaces with different substrate stiffness. We show a facile strategy that allows the fabrication of stable, high resolution patterns for microfluidics and culture of cells over large areas (several cm). Areas covered by PEG-DA are used to prevent non-specific adhesion and confine the cells to spatially defined microstructural features. We demonstrate fibroblast attachment to these patterns and show homogenous and stable cell adhesion and growth over macroscales that can bring microfabrication a step closer to mass production over larger scales for biomedical applications.

## Results and discussion

Non-specific adsorption of proteins on surfaces is a common problem with various biomedical devices such as biosensors, microfluidic devices, and microarrays. Despite a host of favorable properties including flexibility, tunable mechanical properties and oxygen permeability, PDMS surfaces have required surface modification owing to a hydrophobic nature and such non-specific adsorption [11, 31]. A commonly used method for blocking the adsorption of proteins involves immobilizing hydrophilic and neutrally charged polymers to protect the surface [31]. Strategies to prevent protein adsorption can also be used to spatially corral cells on to modified PDMS surfaces [13]. In particular, immobilization of poly (ethylene glycol) (PEG) on surfaces has been widely adopted [21]. Using acrylate functionalized PEGs further allows the integration of microfabrication via photolithography on such surfaces [32]. In this work, micropatterns of PEG-DA were covalently attached to PDMS surfaces as a means to physically control the spatial positioning of cells at the microscale. Since the PEG regions are resistant to the non-specific adhesion of cells, cell growth is confined to exposed regions of the PDMS which in turn, can be functionalized as desired.

### Microchannels of PEG-DA on PDMS surfaces

**Figure 1:**
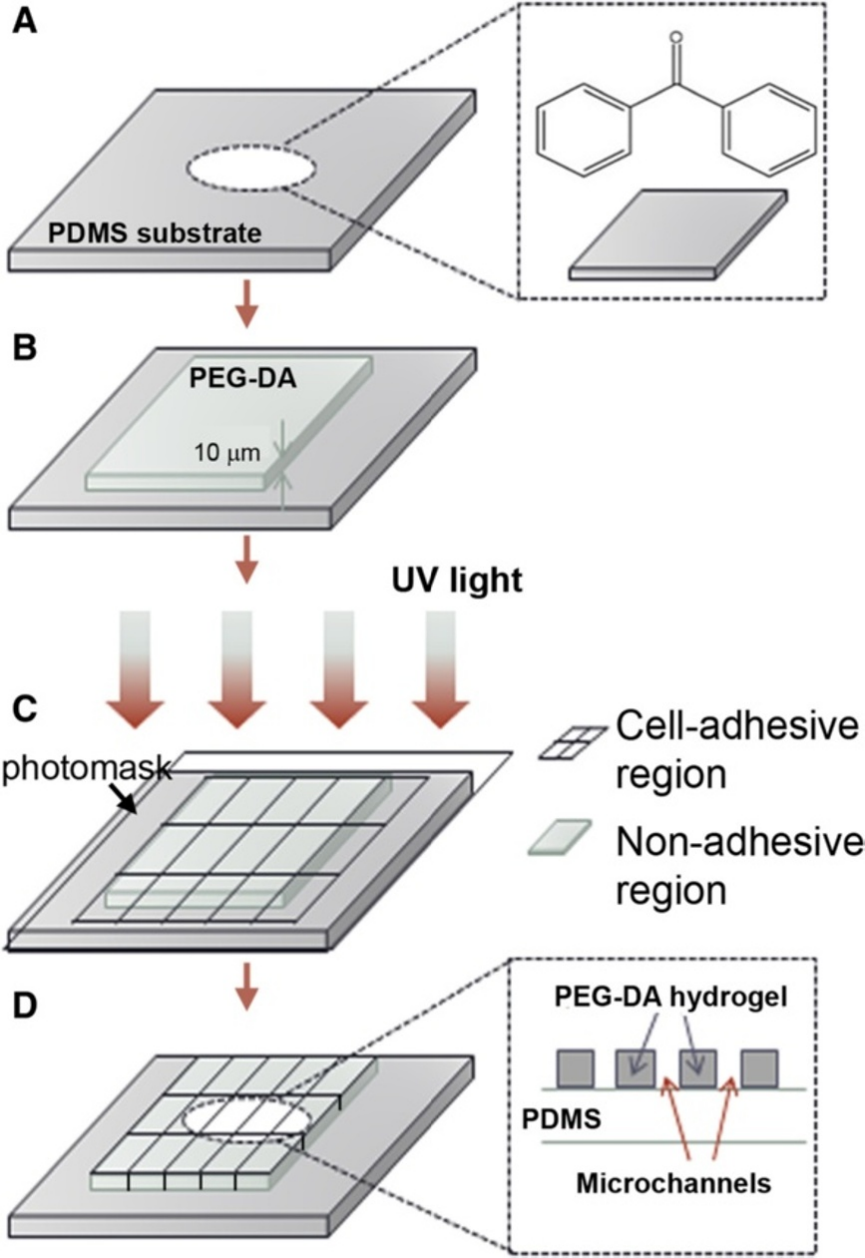
**Schematic of micropatterning on PDMS substrates. (A)** Benzophenone diffusion on to PDMS surface. **(B)** A PEG-DA layer is initially spin coated on the surface (shown to different scale for clarity). **(C)** Exposure of UV through bright field mask. **(D)** Hydrophilic hydrogel micropatterns constructed on the substrate for cells.

PDMS consists of a precursor containing dimethylsiloxane oligomers with vinyl-terminated end groups, mixed with a curing agent containing a crosslinking agent and an inhibitor. Upon crosslinking, the oligomers undergo hydrosilylation and form a Si-C bond [11]. Several studies have reported on microcontact printing (μCP) as a means to pattern cell adhesive moieties on PDMS down to micro and even nanoscales [19, 33, 34]. However questions regarding resolution over large areas, or stability under complex environmental or mechanical cues indicate that these methods may not be optimal for long-term cellular or high-throughput studies. Here PEG polymers which behave as a negative photoresist are covalently grafted on PDMS via UV photopolymerization [25, 35]. This is achieved by first creating active locations for grafting by forming free radicals, followed by covalent attachment of the photocrosslinkable PEG chains to the activated locations. Benzophenone has been previously mixed into the PDMS pre-polymer to form a photoreactive version of PDMS [36]. Here the benzophenone acts as a photoinitiator to aid in the grafting of the monomer. Uncrosslinked PEG-DA following UV exposure through a photomask can then be washed away (developing step) (Figure 1).

**Figure 2:**
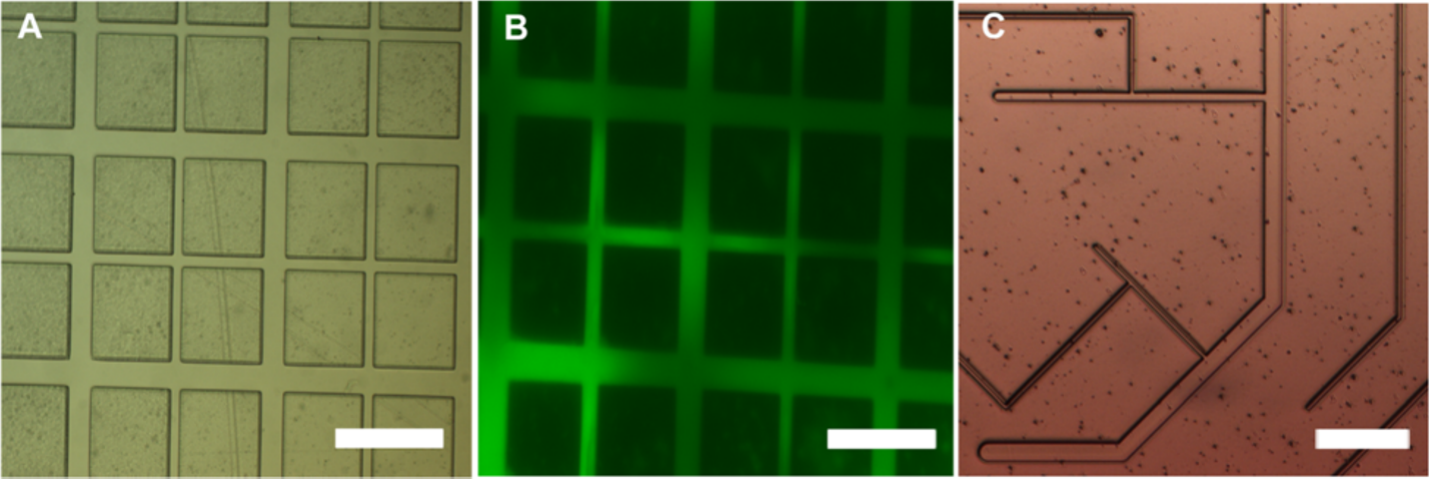
**Micropatterns of PEG on PDMS substrates. (A)** Optical image of micropatterned channels on 10:1 PDMS with 25 and 50 μm width with 150 μm PEG-DA squares. Selective FITC-BSA adhesion to the channels signified by the green fluorescence and resistance to adhesion by the dark PEG-DA 150 μm squares in **(B) (C)** Different patterns with features down to 10 μm can be easily patterned via graft polymerization and photolithography (Scale bar = 200 μm).

Figure 2A shows large scale patterning of 25 and 50 μm channel features on PDMS, reaching an overall area of 2.25 cm^2^ while maintaining their fidelity uniformly throughout the substrate. Squares with sides of 150 μm are regions of PEG-DA hydrogel to limit protein and cell adhesion [20, 24, 37]. The reproducibility of such qualities is vital towards testing selective cell adhesion and growth. This technique can be easily extended to patterns of varying complexity and feature resolutions down to a few micron (Figure 2C). To show that the patterned channels were well developed and reaching the underlying PDMS surface, protein adsorption was tested using fluorescein-labeled albumin. As shown in Figure 2B, the green fluorescence in the channels shows patterned protein adhesion to the channels but not on the PEG-DA squares, signifying specific protein adsorption to the PDMS. Further, it verifies the absence of PEG-DA (due to potential overexposure at the bottom of the channel network) that might hinder protein attachment. This is vital to ensure cell adhesion which would be otherwise impeded by PEG-DA residue in the channels.

### Optimization of micropatterning and stability

The overall strategy focused on two primary objectives – i) high fidelity of micro-architectures over a large (cm) area and, ii) stability of the covalently bound PEG microstructures to permit long-term cell culture. The covalent attachment of PEG to the underlying PDMS was developed using modifications of the methods described earlier [10, 35]. In order to form stable patterns over large areas, several parameters had to be optimized including intensity of the UV light source and time of exposure, which contribute to the amount of light energy transferred to the substrate. Over or under-development during photolithography can result in the formation of cloudy hydrogels, or microstructures that delaminate from the PDMS within a few hours. One successful modification used involves brief intervals between UV exposures that allows heat to dissipate and prevent possible thermal polymerization. In addition, the concentration of the photoinitiator benzophenone, and the chain transfer agent benzyl alcohol are also modulated. Benzyl alcohol aids in the diffusion of the reactive monomers to PDMS surface by decreasing the solution viscosity [31, 35]. This facilitates a stable attachment of the hydrogel to the underlying PDMS which can otherwise delaminate given the large areas. Hydrogels were examined for stability by incubating in a water bath at 37°C over a period of 2 weeks. No delamination was observed and the hydrogels remained intact with the channels maintaining their width. Samples were also observed to be stable over a month when stored dry and rehydrated, showing that this is a robust method. (Image shown in the Additional file 1: Figure S1).

### Removal of benzophenone post-patterning

**Figure 3:**
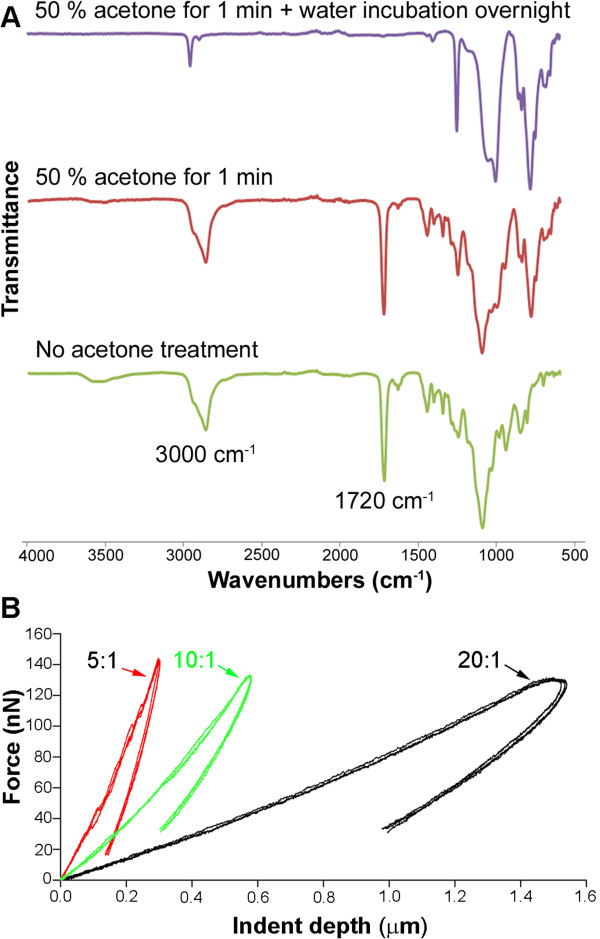
**Surface characterization and mechanical property measurement. (A)** Observation of peaks at 3000 and 1720 cm^-1^ indicates that benzophenone is washed away upon rinsing PDMS with 50 wt% acetone solution for 1 minute and overnight incubation in water (top). Washing with acetone for 1 minute (middle) and untreated PDMS (lower) are shown for comparison **(B)** Sample loading curves of nanoindentation measurements on PDMS. The 20:1 (base:crosslinker ratio) shows more compliant surfaces in comparison to the stiffer 10:1 and 5:1 samples.

A potential concern for cell toxicity is the presence of residual photoinitiator benzophenone following UV polymerization [9, 38]. Further, it was observed that increasing benzophenone exposure beyond 2 minutes results in cloudy suspensions and decreases the stability of the hydrogel. In an earlier work, it was shown that the concentration of benzophenone could be increased to 5% but excess causes the mixture to crystallize. This was also observed in our studies with decreased hydrogel stability [39]. Our reported approach using a low starting concentration of benzophenone ensured that residual photoinitiator was removed prior to cell culture. Immersion of PDMS in acetone was previously suggested as a means to remove benzophenone [10]. However, such treatment resulted in dry hydrogels that delaminate instantly. In our experiments, immersion in a 50 wt. % acetone solution was used, followed by incubation in water overnight and extensive washing. To confirm that the photoinitiator was completely removed, ATR-IR spectroscopy was used to verify the extent of removal. Figure 3 shows surface analyses of PDMS samples for benzophenone following photopolymerization and both conventional and modified acetone treatments. This is monitored by observing peaks near 3000 cm^-1^ representing the C-H stretching of an aromatic ring and the carbonyl group (bridging the two phenyl rings) peak at 1720 cm^-1^. Upon rinsing the samples with the 50% acetone solution and storing them in water overnight, an attenuation of these peaks shows the benzophenone is completely removed. The untreated PDMS is shown as a comparison in the lower trace.

### Effect of substrate stiffness

Cell behavior in terms of proliferation, spreading and attachment can be regulated by altering the stiffness of the substrate. Acting as the *in vitro* extra cellular matrix (ECM), the mechanical properties influence the chemical and physical cues responsible for cell fate. Diverse cell types vary in terms of adhesion and proliferation to changes in stiffness of the substrate. For instance, neural progenitor cells were found to favor neuron and astrocyte differentiation on softer surfaces, but oligodendrocyte differentiation on stiffer substrates [40]. This indicates that such change in the mechanical properties of the substrate can also influence lineage specification. One of the primary advantages of PDMS over substrates such as glass and polystyrene is its tunable mechanical nature and flexibility. However, to date, most studies involving changes in stiffness of PDMS used un-patterned surfaces [11, 28, 30]. Here, in order to optimize cell micropatterning for different cell types, underlying substrate stiffness was further tested as an additional cue.

The stiffness of PDMS can be altered by manipulating the base precursor to cross-linker ratio which dictates the quantity of un-crosslinked oligomers [1, 28]. Typically, optimal PDMS, in terms of cross-linking density and amount of un-crosslinked oligomers, is standardized as 10:1 (base: crosslinker). Here, three ratios were tested – 5:1, 10:1 and 20:1 PDMS, where an increase in the amount of crosslinker results in stiffer substrates [41]. Uncrosslinked, low molecular weight oligomers that can influence the surface chemistry and therefore cell behavior on the substrate. Excess curing agent, as in the 5:1 PDMS, means a stiffer substrate since further crosslinking is promoted [42]. Surplus of precursor, as in the 20:1 PDMS, leads to a softer substrate due to insufficient curing agent and the presence of unlinked vinyl terminated oligomers [11]. While temperature and curing time also contribute to the rigidity of PDMS, they were kept constant at 62°C for 24 hours. Table 1 demonstrates the variance in the Young’s modulus as obtained using AFM-based nanoindentation. As expected, increasing the ratio of crosslinker to precursor causes an increase in the modulus and stiffness [43] (Figure 3B). The micropatterning strategy developed above is versatile enough to be adaptable to all stiffness of PDMS without any significant change in protocol. Typically the different PDMS substrates required adjustments in only the time of UV exposure. Images showing protein adsorption to channels of 5:1 and 20:1 PDMS is presented in the Additional file 1: Figure S2.

**Table 1:** **Elastic moduli and stiffness of PDMS with varying base to curing agent ratios**

Base: CA	Modulus (MPa)	Stiffness (N/m)
5:1	6.10 ± 0.11	1.90 ± 0.04
10:1	2.95 ± 0.05	1.35 ± 0.02
20:1	1.38 ± 0.05	1.10 ± 0.04

### Cell culture on patterned PDMS surfaces

**Figure 4:**
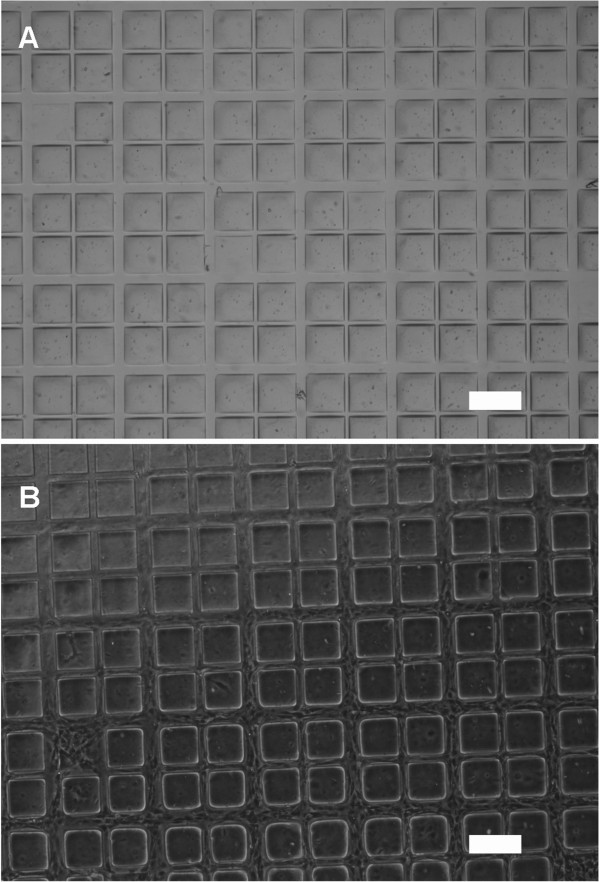
**Patterning of fibroblasts. (A)** Large scale micropatterning of PEG-DA by photografting on PDMS. **(B)** Mouse 3T3 fibroblasts grown on the patterns. Features had high fidelity over the total area of the micropatterns ~1.5 × 1.5 cm.

**Figure 5:**
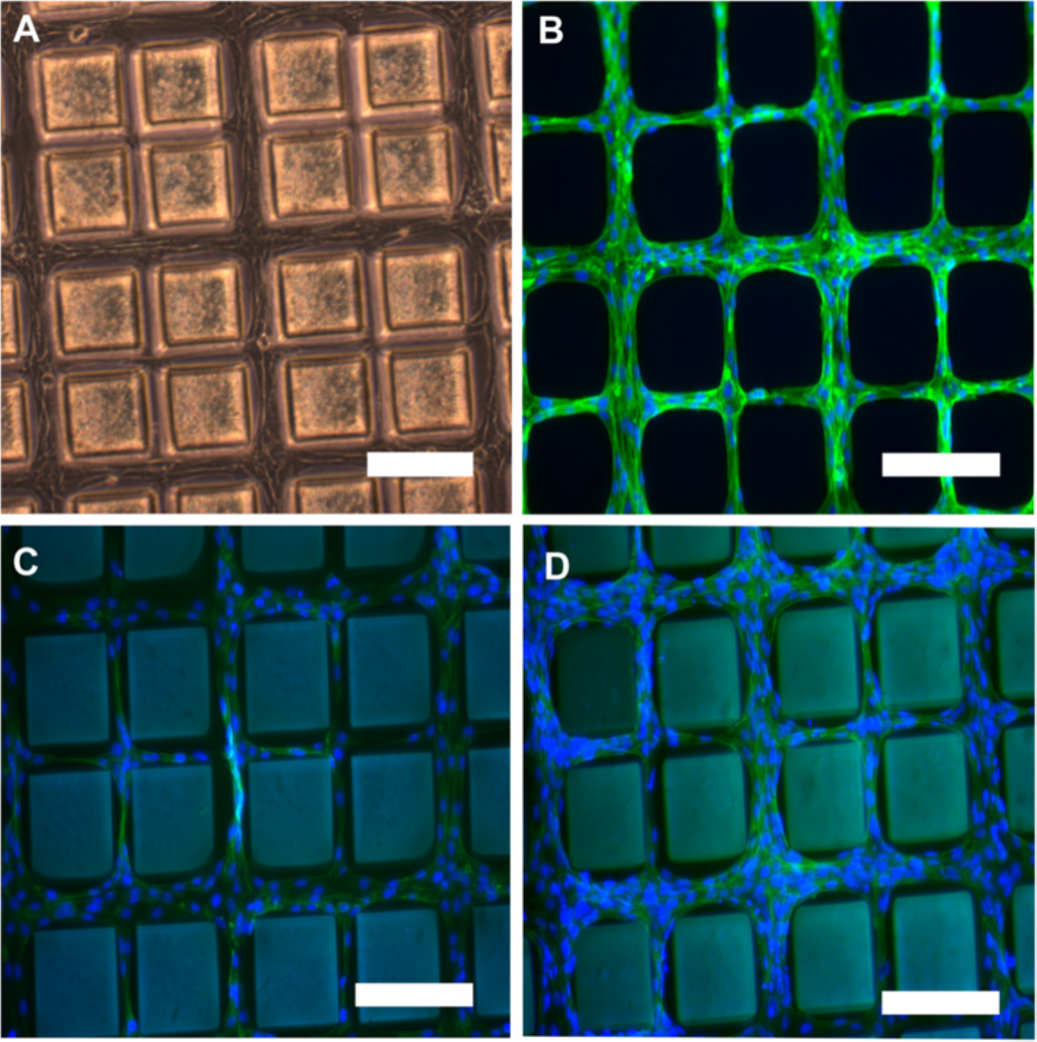
**Effect of substrate stiffness. (A)** A bright field optical image of human dermal fibroblasts after 6 days on 10:1 PDMS forming an interconnected network around 150 μm squares of PEG-DA**.** Phalloidin and DAPI stained cells on **(B)** 10:1 PDMS **(C)** 20:1 PDMS and **(D)** 5:1 PDMS shown with the nuclei and cytoskeleton overlaid. (Scale bar on all images = 200 μm).

The patterned surfaces were studied for cell adhesion and growth with a focus on cell avoidance to the PEG regions and confinement to PDMS. Since native PDMS does not promote cell attachment due to its high hydrophobicity [12], coating the PDMS with fibronectin allowed cell adhesion and proliferation. In our experiments, two cell lines - 3T3 mice fibroblasts and human dermal fibroblasts (HDFs) were tested at a density of 5×10^4^ cells/well on fibronectin coated PDMS samples. After 6 days, the PEG-DA hydrogel regions showed no cell attachment as expected. The cells were shown to be fully proliferated and interconnected at high density throughout the microchannel patterns to form a network over a large area (Figure 4). Here we show representative images of each culture. The 50 μm channels showed the aggregation of several cells compared to the smaller 25 μm ones which had only a few cells aligned. This indicated that not only was the adhesion confined to the PDMS channels, but that the cells responded to spatial cues and distributed accordingly. Consistent proliferation of the cells throughout the pattern was also achieved over a large area (typically 1–2.5 cm) indicating that this is a scalable method that can be used to cover an area such as a tissue culture well or petridish. The cells were fixed using 4% paraformaldehyde in PBS and stained with both DAPI and phalloidin. Figure 5B shows fluorescence imaging of the cells with the nuclear and cytoskeleton stains overlaid.

Earlier, a higher adhesion density of 3T3 mouse fibroblasts on un-patterned 20:1 PDMS than the stiffer 5:1 and 10:1 surfaces had been reported. Despite being less stiff at the macroscale, 20:1 PDMS is more pliable at the nanoscale resulting in exposed cell-binding motifs [30]. On the other hand, a different study found transformed fibroblasts to grow at similar rates on PDMS irrespective of stiffness [11]. In our experiments, expectedly, the morphology and slow proliferation of the adhered cells is an indication that substrate stiffness is an important contributor in micropatterned samples as well. This is observed in Figure 3 which shows consistent fidelity of the features across samples but lower cell density. Both 5:1 and 20:1 PDMS maintained precise cell adhesion to the channels but at lower densities compared to the 10:1 samples (Figure 5c, d). Favoring 10:1 PDMS over 20:1 supports the claim that fibroblasts may migrate preferentially to stiffer substrates and exhibit stronger traction forces [44]. Similarly, the cell proliferation on stiffer 5:1 PDMS was higher in comparison to 20:1 PDMS. Overall this could be attributed to the optimal cross-linking density of 10:1 PDMS and the higher amount of un-crosslinked components when increasing the cross-linker or based beyond the normal ratio [11]. These components can be either mobile affecting the nutrients in growth media or stationary on the PDMS surface influencing cell attachment and growth. However, since softer substrates are desirable for certain cultures, it is important to note that overall, the formation of high-resolution PEG microstructures on PDMS of different stiffness demonstrates that this strategy can be adapted to substrates of tunable mechanical properties to control cell growth.

## Conclusions

The integration of microfabrication and cell culture can result in efficient and directed cellular responses with uniform cell patterning over large length scales. In this study, we demonstrate a technique for the fabrication of stable and long-lasting PEG-DA hydrogels that are photografted on PDMS of varying stiffness. The resultant micropatterns can be formed at a high resolution down to 5–10 μm. The patterns are robust over a period of weeks and maintain function in cell culture medium, specifically resisting cell adhesion and directing cell growth. By increasing benzyl alcohol concentration in the PEG-DA monomer, decreasing benzophenone treatment and using intermittent UV exposure, both hydrogel attachment and resolution were optimized. Such measures further lead to specific, high density protein and cell adhesion on PDMS that was uniformly micropatterned up to an area of 2–4 cm^2^. The effect of altering the mechanical properties of the patterned substrate, through stiffness, on cell behavior was examined on 5:1, 10:1 and 20:1 PDMS samples. Controlled cell adhesion and proliferation was achieved for all three ratios with the cells migrating specifically to the PDMS channels surrounded by PEG-DA. 10:1 PDMS, which is very commonly used, was also shown to be a versatile and flexible surface in terms of growing fibroblasts on micropatterned regions. The ability to further functionalize and tune the PDMS surface and its underlying stiffness, provides a method to tailor the attachment and culture of various cells in different geometries. This study shows potential to further increase the micropatterning areas while reaching smaller widths on the microscale. More complex features can also be adapted that mimic the *in vivo* microenvironment and be used for directing cells into specific lineages and controlling their fate.

## Methods

### Materials

Poly (dimethyl siloxane) (PDMS) prepolymer and curing agent (Sylgard 184) were obtained from Dow Corning (Midland, MI). PEG-DA (number average molecular weight, 575) and albumin-fluorescein isothiocyanate conjugate were purchased from Sigma-Aldrich (St. Louis, MO). Benzophenone, acetone, Benzyl alcohol, Sodium periodate (NaIO_4_), methanol, paraformaldehyde powder and Phosphate buffered saline (PBS) solution (10X) were obtained from Fisher Scientific (Fair Lawn, NJ) and used as received. 3T3 mice fibroblasts and human dermal fibroblasts were used to test cell adhesion and cell growth. 4′, 6-diamidino-2-phenylindole (DAPI) and Alexa Fluor Phalloidin 488 (Life Technologies, Grand Island, NY) were used for staining. A bright-field reflective chrome photomask was designed using CleWin and custom fabricated to form large area grids consisting of 50 and 25 μm lines.

### UV photografting of PEG-DA on PDMS

To prepare the PDMS substrates of varying stiffness, the mass ratio of base to curing agent was varied to form (in order of decreasing stiffness) 5:1, 10:1 and 20:1 samples. 12.5 g of pre-polymer was mixed with 1.25 g, 0.625 g and 2.5 g of curing agent respectively and added to a 60 mm plastic petridish. After overnight curing at 62°C, the PDMS was peeled off and diced into squares (~1-4 cm^2^). PDMS slabs were then immersed in a 10 wt. % benzophenone solution in acetone for 2 minutes. The samples were rinsed with methanol and air dried.

1 ml of 40wt% PEG-DA solution was prepared by dissolving 400 μl of PEG-DA, 10 μl of 100mM NaIO_4_ (1 mM) and 50 μl of benzyl alcohol (5 wt. %) in water. 65 μl of the reaction solution was cast on the PDMS surfaces. PEG-DA was photopolymerized through a photomask using a 365 nm, 500 mW/cm^2^ light source (OmniCure S1000, Lumen Dynamics). PEG-DA behaves as a negative photoresist in the presence of UV light and is crosslinked owing to the photoinitiator benzophenone [32, 45]. The schematic for this reaction is shown in the (Additional file 1: Figure S4). The exposure conditions were optimized by varying intensity and time of exposure to obtain large patterned areas. Figure  1 shows a schematic of the steps involved in obtaining a stable, micropatterned PEG-DA hydrogel on PDMS. Micropatterned PDMS samples were stored in 1x PBS and sterilized by UV exposure for 30 minutes (<0.01 W/cm^2^) prior to cell studies. To promote cell attachment, samples were subsequently immersed in 3 ml of 6 μg/ml fibronectin solution for one hour prior to cell culture.

### Cell culture on micropatterned samples

3T3 mice fibroblasts were cultured at 37°C in 5% humidified environment in Dulbecco’s modified eagle’s medium (DMEM) with 10% fetal bovine serum. On reaching 90% confluence in four days, the cells were trypsinized (0.25% trypsin solution) and passaged at a density of 5×10^4^ cells per PDMS sample. Human dermal fibroblasts (HDFs) were maintained in minimum essential medium supplemented with 2 mM l-glutamine, 1% penicillin/streptomycin, 15% fetal bovine serum, non-essential and essential amino acids, sodium pyruvate and vitamins [46]. Cells were cultured on the 5:1 and 20:1 samples at the same density. For cell staining, PDMS samples were each covered with 1 mg/ml of albumin-fluorescein isothiocyanate (FITC-BSA) conjugate for 30 minutes before being examined under a fluorescence microscope (Nikon ECLIPSE TE2000-U). Following 6 days of cell culture, the samples were fixed by adding 4% paraformaldehyde solution for 30 minutes. Phalloidin 488 was added as a cytoskeletal stain and DAPI as a nuclear stain for 30 minutes each. The samples were washed 3 times for 5 minutes each using PBS wash buffer following each of the fixing and staining steps.

### Nanomechanical measurements of PDMS substrates

Mechanical properties of crosslinked films of PDMS were measured using AFM-based nanoindentation (MFP-3D, Asylum Research, Santa Barbara, CA). All samples were indented using an AC160 TS cantilever (Olympus Research, Tokyo, Japan) with nominal spring constants varying from 30–40 N/m. The actual spring constants were determined prior to each experiment using the thermal fluctuation method on a hard mica surface [47]. Different PDMS samples were indented in air with ~30 indents at different areas on the surface using constant force mode (100 nN). The Young’s modulus and stiffness were obtained via the Oliver-Pharr model in Igor Pro 6.22 A (Wavemetrics Inc., OR) [48] .

## Electronic supplementary material

Additional file 1: Figure S1: Micropatterned PEG-DA squares on PDMS after 2 months storage. Samples could be rehydrated and used for cell culture. **Figure S2.** Large scale grids formed on PDMS. Squares are 150 μm PEG-DA surrounded by 25 and 50 μm channels. Adsorption experiment with FITC-BSA on the different substrates showing the non-specific adsorption to the PEG is minimal with the protein attaching to the PDMS. **Figure S3.** Large area micropatterning of cells on PDMS. Area on the left shows a PEG-DA square that had delaminated. Cells reach the underlying PDMS substrate and begin to proliferate. **Figure S4.** Schematic showing the chemistry of photo-induced polymerization of PEG-DA in the presence of benzophenone as the photoinitiator and UV light irradiation. The PEG-DA behaves as a negative photoresist and crosslinks in the presence of UV light turning from liquid to solid in the process. (*adapted from C. Decker – “Photoinitiated crosslinking polymerization”, Prog. Polym. Sci. 21, 593-650, 1996*). (DOCX 3 MB)
