# Supplementary material for: Large area micropatterning of cells on polydimethylsiloxane surfaces
Source: J Biol Eng. 2014 Oct 24;8:24. doi: 10.1186/1754-1611-8-24 (PMC4223844; doi:10.1186/1754-1611-8-24)
Supplement: Supplementary file 1 — Additional file 1: Figure S1: Micropatterned PEG-DA squares on PDMS after 2 months storage. Samples could be rehydrated and used for cell culture. Figure S2. Large scale grids formed on PDMS. Squares are 150 μm PEG-DA surrounded by 25 and 50 μm channels. Adsorption experiment with FITC-BSA on the different substrates showing the non-specific adsorption to the PEG is minimal with the protein attaching to the PDMS. Figure S3. Large area micropatterning of cells on PDMS. Area on the left shows a PEG-DA square that had delaminated. Cells reach the underlying PDMS substrate and begin to proliferate. Figure S4. Schematic showing the chemistry of photo-induced polymerization of PEG-DA in the presence of benzophenone as the photoinitiator and UV light irradiation. The PEG-DA behaves as a negative photoresist and crosslinks in the presence of UV light turning from liquid to solid in the process. (adapted from C. Decker – “Photoinitiated crosslinking polymerization”, Prog. Polym. Sci. 21, 593-650, 1996). (DOCX 3 MB) [file 13036_2014_156_MOESM1_ESM.docx]

**Additional file 1:**

**Large area micropatterning of cells on polydimethylsiloxane surfaces**

Mahmoud E. Moustafa, Venkat S. Gadepalli, Ahmed A. Elmak, Woomin Lee, Raj R. Rao, Vamsi K. Yadavalli ^*^

Department of Chemical and Life Science Engineering, Virginia Commonwealth University,

Richmond VA 23284, USA

Corresponding author:

^*^ Dr. Vamsi K. Yadavalli

Department of Chemical and Life Science Engineering,

Virginia Commonwealth University,

Richmond VA 23284, USA

Phone: 1-804-828-0587 Fax: 1-804-828-3846

Email: [vyadavalli@vcu.edu](mailto:vyadavalli@vcu.edu)


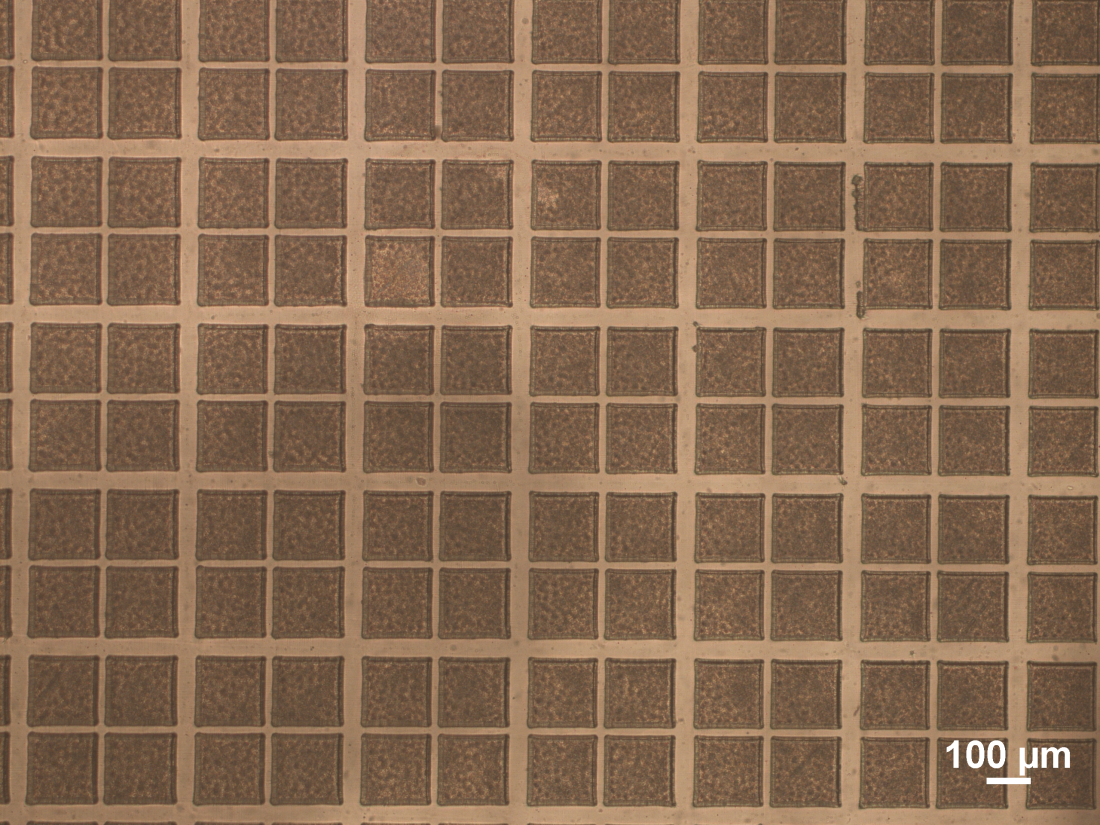


**Figure S1:** Micropatterned PEG-DA squares on PDMS after 2 months storage. Samples could be rehydrated and used for cell culture.


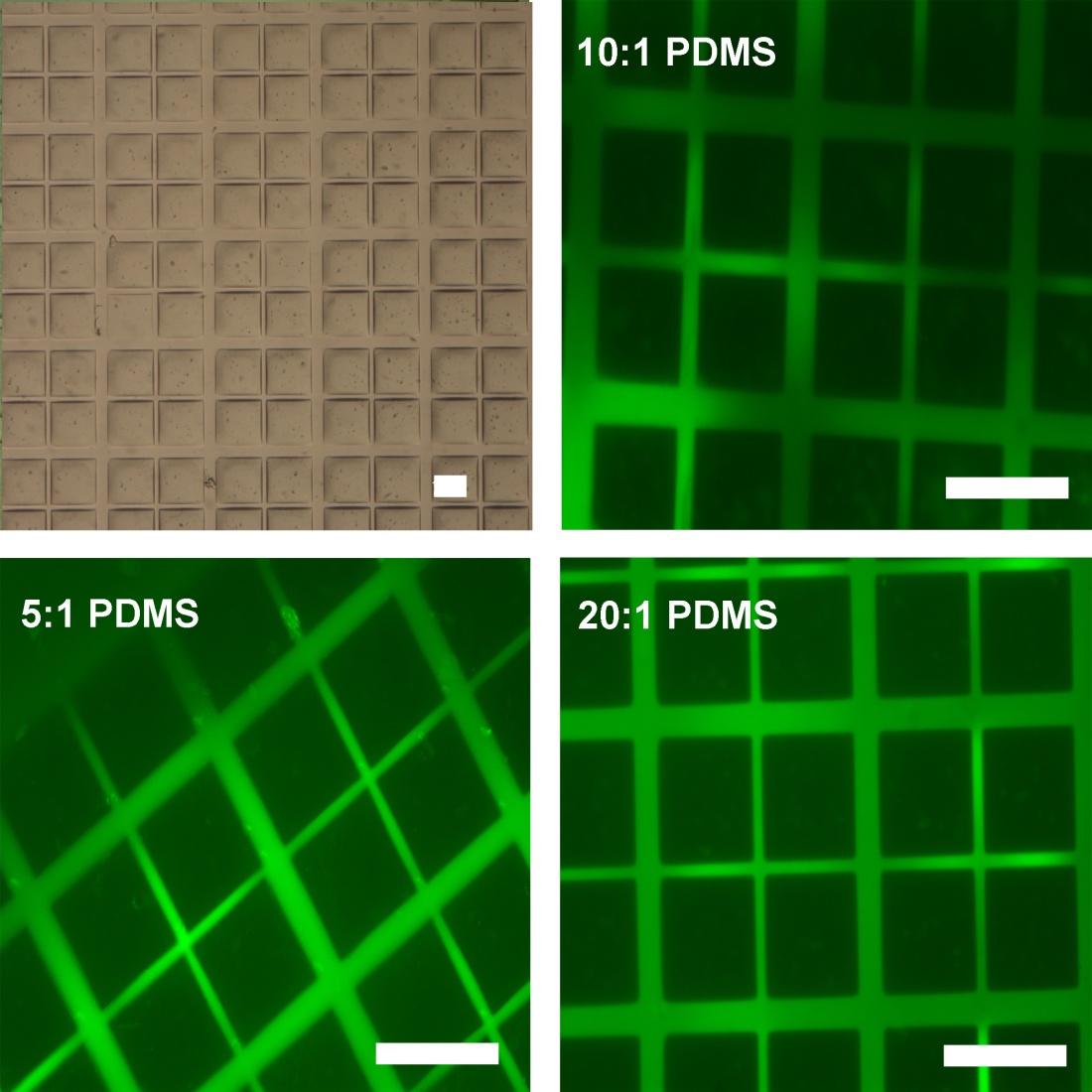


**Figure S2**: Large scale grids formed on PDMS. Squares are 150 µm PEG-DA surrounded by 25 and 50 µm channels. Adsorption experiment with FITC-BSA on the different substrates showing the non-specific adsorption to the PEG is minimal with the protein attaching to the PDMS.


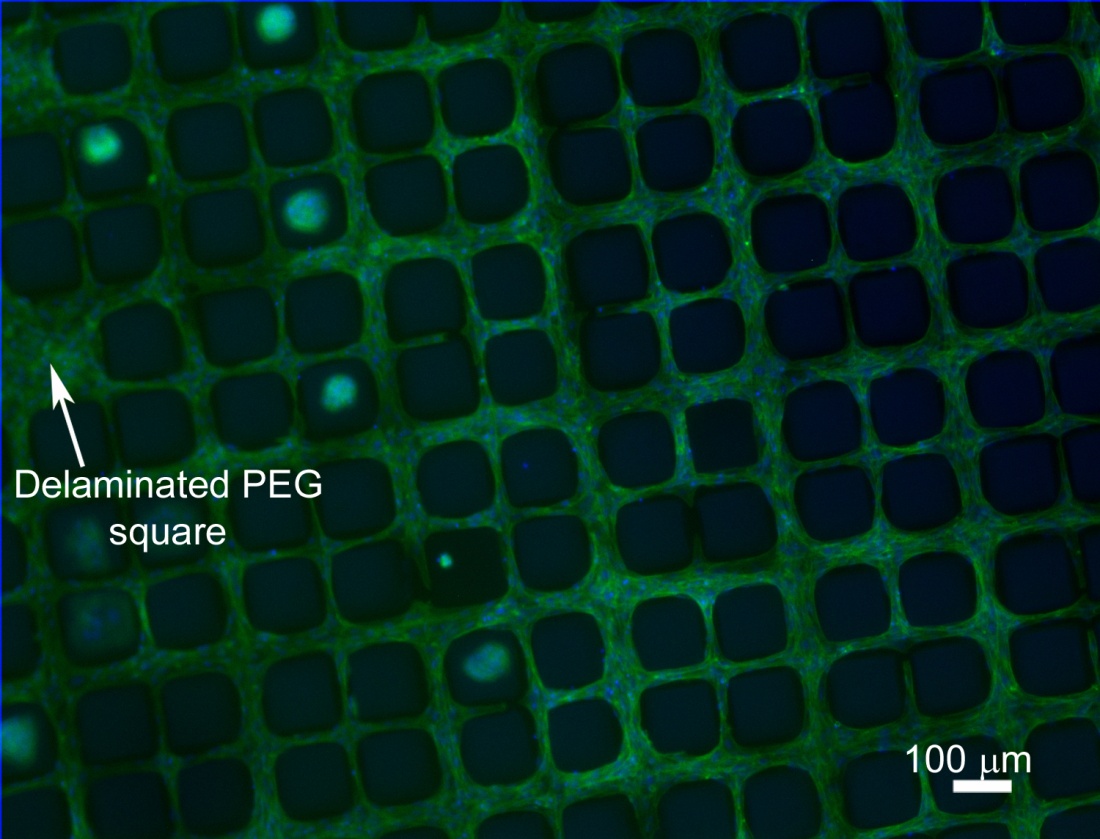


**Figure S3**: Large area micropatterning of cells on PDMS. Area on the left shows a PEG-DA square that had delaminated. Cells reach the underlying PDMS substrate and begin to proliferate.


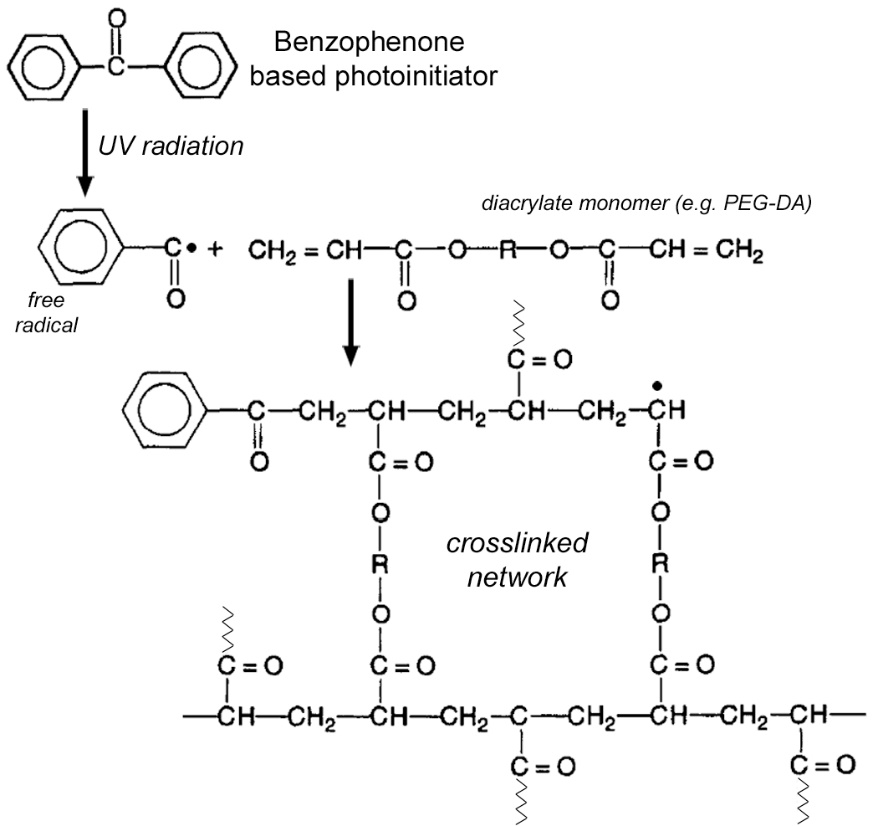


**Figure S4:** Schematic showing the chemistry of photo-induced polymerization of PEG-DA in the presence of benzophenone as the photoinitiator and UV light irradiation. The PEG-DA behaves as a negative photoresist and crosslinks in the presence of UV light turning from liquid to solid in the process. (*adapted from C. Decker – “Photoinitiated crosslinking polymerization” , Prog. Polym. Sci. 21, 593-650, 1996*)
